# The Use of Kidney Disease Improving Global Outcomes AKI Definitions in AKI Research: PRO

**DOI:** 10.34067/KID.0000000000000486

**Published:** 2024-07-25

**Authors:** Ian E. McCoy, Glenn M. Chertow

**Affiliations:** 1 Division of Nephrology, University of California San Francisco, San Francisco, California; 2 Departments of Medicine, Epidemiology and Population Health, and Health Policy, Stanford University School of Medicine, Stanford, California

**Keywords:** AKI

AKI has been defined in a myriad of ways, including those on the basis of changes in serum creatinine, urine output, and a host of putative biomarkers. While in some sense this range of definitions is problematic (*e.g*., complicating comparison of results from different studies), different AKI definitions may be appropriate for different purposes. For instance, a highly sensitive, nonspecific definition like the 0.3 mg/dl absolute increase in serum creatinine incorporated within the 2012 Kidney Disease Improving Global Outcomes (KDIGO) AKI criteria^1^ may be appropriate for use in exploratory analyses of AKI as an exposure in epidemiological studies evaluating patient-centered outcomes (*e.g*., in-hospital mortality, persistent decrements in kidney function at 90 days). By contrast, it would be inappropriate to use the 0.3 mg/dl increase in serum creatinine definition as a safety endpoint in a clinical trial^2^ (Figure 1). Indeed, episodes of mild (*e.g*., KDIGO stage 1) AKI have been paradoxically associated with favorable outcomes in numerous studies,^2^ including the recent Dapagliflozin and Prevention of Adverse Outcomes in CKD trial.^3^

**Figure 1:**
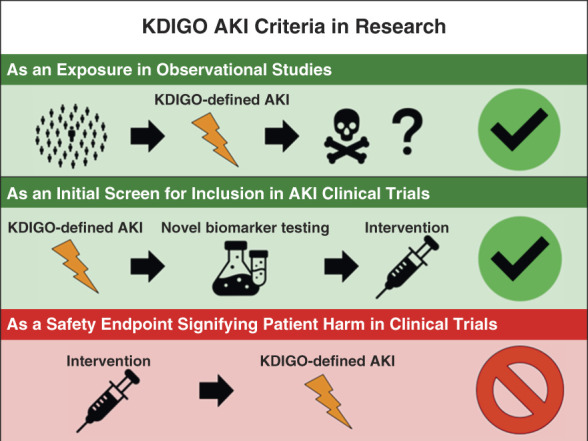
**Suggested uses of KDIGO criteria in AKI research.** KDIGO, Kidney Disease Improving Global Outcomes.

With that caveat aside, we argue here that the KDIGO AKI criteria are the best currently available for use in many aspects of AKI research.

## The KDIGO AKI Criteria Associate with Adverse Outcomes

The KDIGO AKI criteria bring consensus to the AKI literature, allowing comparison of AKI associations in various patient populations and clinical contexts. Iterating on the 2004 Risk, Injury, Failure, Loss, and End-stage kidney disease criteria put forth by the Acute Dialysis Quality Initiative, the 2012 KDIGO criteria added the stage 1 AKI definition of a 0.3 mg/dl increase in serum creatinine within 48 hours to the prior stage 1 AKI definition of an increase in serum creatinine to 1.5× the baseline level within 7 days. Acute increases in serum creatinine as small as 0.3 mg/dl have been consistently linked to hospital mortality,^4,5^ as well as lengths of stay and costs.^4^ The independent association between the 2012 KDIGO AKI criteria (and stage definitions) and mortality has been validated in patient cohorts worldwide.^5^

## The KDIGO AKI Criteria Can Be Applied to Routinely Available Clinical Data

Serum creatinine is included in the basic metabolic panel, measured in most hospitalized patients.^6^ The KDIGO serum creatinine criteria allow AKI status to be ascertained from data routinely available in the electronic health record. The inclusion of an absolute increase in serum creatinine definition (≥0.3 mg/dl within 48 hours) allows for AKI ascertainment among patients without an available baseline serum creatinine, mitigating the need for the inelegant and potentially incorrect back-calculation of a baseline serum creatinine that assumes a normal eGFR.

The KDIGO AKI criteria can also be used to seamlessly screen patients for inclusion in AKI-related clinical trials. As novel biomarkers emerge to facilitate phenotyping of AKI, such as CXCL9 for acute interstitial nephritis,^7^ investigators may wish to initially screen for clinical trial eligibility using the KDIGO criteria, and then refine eligibility with a more specific biomarker measured in sequence (Figure 1).

The KDIGO criteria also incorporate urine output into the stage definitions, which in some contexts could allow for earlier detection of AKI before sufficient time has passed to yield a change in serum creatinine. However, the urine output criteria are less often used in research because of the effort required to collect and measure hourly urine output, innumerable confounding factors (*e.g*., comorbid conditions, administration of crystalloids, colloids, and diuretics, *etc.*), and less consistent associations with outcomes.

## The KDIGO AKI Criteria Include Stages, Allowing Flexibility for Different Applications

Although the KDIGO criteria are often used to ascertain AKI status as a binary variable (*i.e*., meeting *any* criteria for any stage of AKI versus *no* criteria for any stage of AKI), the KDIGO criteria incorporate distinct definitions for AKI stages 1, 2, and 3, recognizing increasingly severe AKI episodes. AKI stages predict incrementally worse outcomes and thus can be useful to select populations at different levels of illness severity. The AKI stages allow the researcher to choose to include all AKI stages when aiming to maximize sensitivity, while restricting to stage 2 and/or 3 AKI in contexts where the balance weighs in favor of specificity (*e.g*., validation of novel AKI biomarkers^8^ or an invasive or higher-risk intervention).

## The KDIGO AKI Criteria are Reproducible

The KDIGO AKI criteria are objective, quantitative criteria that can be reproduced from analyst to analyst and study to study. Modern enzymatic assays for serum creatinine have resulted in improved precision over older Jaffe assays. Furthermore, a decades-long effort has resulted in the standardization of serum creatinine measurement to isotope dilution mass spectrometry, which allows for accurate comparison between different laboratories (often distinct for measurements of the baseline serum creatinine in the outpatient setting and of the higher serum creatinine value defining AKI in the hospital).^9^ Once newer biomarkers of kidney function become commonplace, it is likely that a similar effort will be needed to maximize precision and achieve standardization in each laboratory and across laboratories.

In conclusion, the KDIGO AKI criteria represent a reproducible, well-validated set of diagnostic definitions that can be easily implemented from routinely available clinical data. Although the KDIGO criteria should not be indiscriminately used in all aspects of AKI research (*e.g*., as a safety endpoint in clinical trials), the KDIGO criteria remain a time-tested and valuable option for use in many aspects of AKI research.
